# Breaking the Algorithm: Unearthing Life-Threatening Multivessel Spontaneous Coronary Artery Dissection (SCAD) After a Negative Observation Unit Workup

**DOI:** 10.7759/cureus.106900

**Published:** 2026-04-12

**Authors:** Meaghan Bethea, Zechariah Jean, Syed Ali, George Dirani, Matthew Boutorwick

**Affiliations:** 1 Emergency Medicine, Ross University School of Medicine, Warren, USA; 2 Emergency Medicine, Henry Ford Health System, Warren, USA

**Keywords:** acute coronary syndrome, cardiac chest pain, coronary artery, dissection, emergency observation unit, etiology, scad diagnosis, spontaneous, young woman

## Abstract

Spontaneous coronary artery dissection (SCAD) is an underrecognized, non-atherosclerotic cause of acute coronary syndrome (ACS) that disproportionately affects younger women. We present the case of a 34-year-old woman with hypertension who presented to the emergency department with acute chest pain, dyspnea, and vomiting, only one week after a reassuring chest pain evaluation in an observation unit. Prehospital electrocardiography demonstrated arrhythmia with premature ventricular contractions, followed by inferior-lateral ST-T changes on subsequent tracings. These acute findings prompted coronary angiography, which confirmed SCAD. This case highlights that SCAD may occur even after a negative observation-unit evaluation and emphasizes the importance of maintaining a high index of suspicion for SCAD in young women presenting with recurrent ischemic symptoms. In addition, it includes the potential limitations of conventional chest pain algorithms in detecting non-atherosclerotic causes of ACS and calls for potential branchpoints in risk stratification pathways to incorporate additional cardiovascular syndromes.

## Introduction

Chest pain is one of the most common emergency department (ED) presentations, and observation-unit pathways incorporating high-sensitivity troponin assays and structured risk scores such as the HEART (history, electrocardiogram, age, risk factors, and troponin) score have significantly improved diagnostic safety and efficiency in evaluating suspected acute coronary syndrome (ACS) [1]. However, these pathways are primarily validated for atherothrombotic ACS and may be less sensitive for alternative causes of myocardial ischemia. Important non-atherosclerotic etiologies, including spontaneous coronary artery dissection (SCAD), may therefore be underrecognized, particularly in younger patients without traditional cardiovascular risk factors [2-4].

SCAD is a non-atherosclerotic cause of ACS characterized by intramural hematoma or intimal disruption leading to coronary luminal narrowing and myocardial ischemia [2-4]. Although historically considered rare, SCAD is now recognized to account for approximately 1%-4% of ACS presentations overall and up to 25% of myocardial infarctions in women younger than 50 years [2-4]. Increasing awareness of SCAD has highlighted important diagnostic challenges, particularly when patients initially present with reassuring electrocardiographic findings and negative serial high-sensitivity troponin testing.

This case describes a 34-year-old woman who developed multivessel SCAD one week after a negative ED observation-unit chest pain evaluation using an accelerated diagnostic pathway. It highlights the limitations of relying solely on structured chest pain algorithms when evaluating recurrent ischemic symptoms in young women and underscores the importance of maintaining clinical suspicion for non-atherosclerotic causes of ACS.

## Case presentation

A 34-year-old woman with a history of hypertension, managed with lisinopril 2.5 mg daily, presented to the ED with acute-onset left anterior chest pressure described as aching and non-radiating. Symptoms began approximately 5-10 minutes before emergency medical services (EMS) arrival and were associated with dyspnea, nausea, and vomiting. She denied prior similar episodes, palpitations, syncope, or known coronary artery disease.

Initial ED encounter (one week prior)

**Figure 1:**
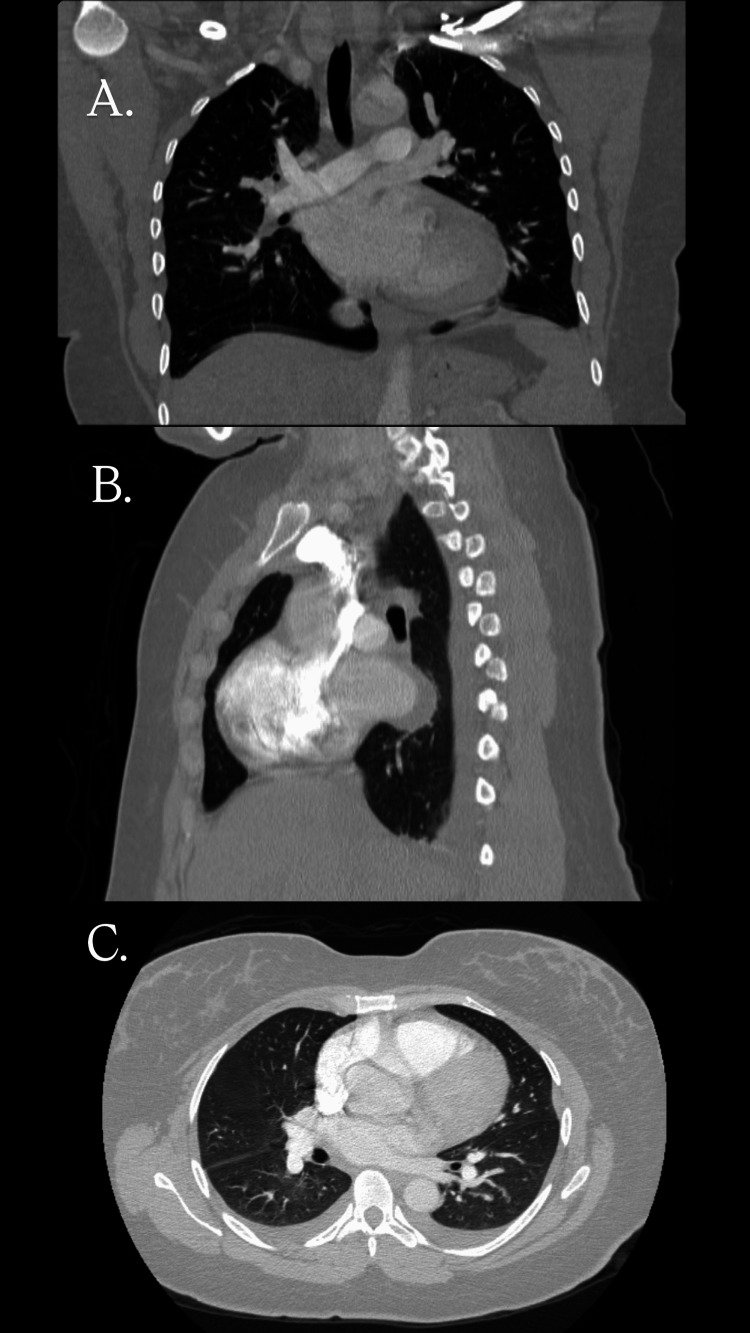
Computed tomography (CT) of the chest with intravenous contrast. (A) Coronal view demonstrating an enlarged cardiac silhouette with mild bilateral pleural effusions. (B) Sagittal view highlighting the enlarged heart relative to the thoracic cavity. (C) Axial lung window image showing small bilateral pleural effusions without focal consolidation. No evidence of pulmonary embolism was identified.

One week earlier, the patient had been evaluated at another ED and admitted to the observation unit for elevated blood pressure and bilateral lower extremity edema. Computed tomography angiography of the chest excluded pulmonary embolism but demonstrated cardiomegaly with small bilateral pleural effusions (Figure 1).

Bilateral lower extremity venous Doppler ultrasonography was negative for deep venous thrombosis. Transthoracic echocardiography demonstrated preserved left ventricular systolic function with an estimated ejection fraction of 60%-65% and no regional wall motion abnormalities or pericardial effusion (Video 1).

**Video 1:** Transthoracic echocardiogram obtained during the patient’s initial observation-unit evaluation demonstrating normal left ventricular systolic function with preserved ejection fraction (~60%-65%) and no regional wall motion abnormalities. Both right and left ventricular chamber sizes appear normal, and there is no pericardial effusion or valvular pathology. Findings are consistent with normal cardiac structure and function prior to the development of spontaneous coronary artery dissection (SCAD).

**Figure 2:**
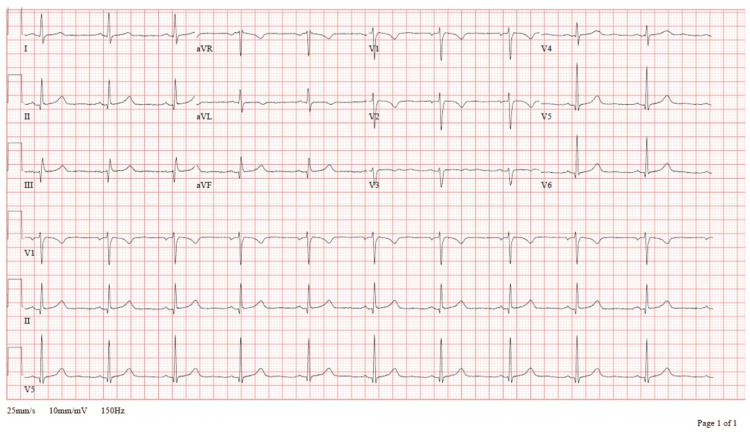
Twelve-lead electrocardiogram obtained one week before presentation demonstrating normal sinus rhythm at 62 beats per minute, with possible inferior and anterior infarct patterns of undetermined age. ST segments are isoelectric without acute elevations or depressions. No dynamic ischemic changes were present compared with subsequent ECGs, which later demonstrated new lateral ST-segment elevations consistent with evolving myocardial ischemia secondary to spontaneous coronary artery dissection (SCAD).

During that admission, serial high-sensitivity troponin testing demonstrated mild elevation without a rising pattern (peak: 16 ng/L; reference: <14 ng/L), followed by downtrending values. Electrocardiography showed a normal sinus rhythm with possible left atrial enlargement and nonspecific ST-segment changes (Figure 2), which cardiology determined were not consistent with ischemia. At the time of cardiology evaluation, the patient denied chest pain and appeared euvolemic. As the patient satisfied institutional criteria for low-risk chest pain using an accelerated diagnostic pathway incorporating serial high-sensitivity troponin testing and electrocardiographic monitoring, additional anatomic coronary imaging, such as coronary CT angiography, was not pursued in accordance with contemporary guideline-supported ED chest pain workflows. She met institutional criteria for low-risk chest pain observation overnight and was discharged home with initiation of lisinopril and outpatient follow-up.

Current presentation

**Figure 3:**
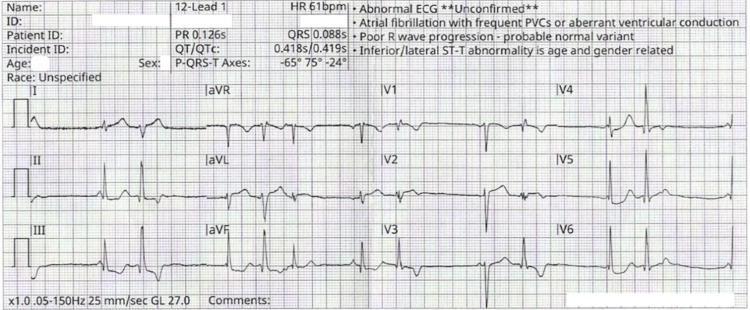
Twelve-lead electrocardiogram obtained by EMS demonstrating sinus rhythm at 61 beats per minute (PR 126 ms, QRS 88 ms, and QTc 419 ms), left axis deviation (−65°), and poor R-wave progression across the precordium. Nonspecific ST-T abnormalities are most pronounced in the inferior (III, aVF) and lateral (I, aVL, V5-V6) leads, including 1-2 mm ST-segment depression in leads III and aVF. Findings could be concerning for myocardial ischemia; however, a repeat ECG en route demonstrated a return to normal sinus rhythm. EMS: Emergency medical services; PVCs: Premature ventricular contractions.

Upon EMS arrival for the current presentation, the patient was in mild distress. Prehospital electrocardiography demonstrated sinus rhythm with frequent premature ventricular contractions and nonspecific ST-T abnormalities in the inferior and lateral leads (Figure 3). She received aspirin and nitroglycerin without symptom relief. A subsequent ECG en route demonstrated a return to sinus rhythm.

In the ED, vital signs included blood pressure of 176/98 mmHg, heart rate of 79 beats per minute, respiratory rate of 22 breaths per minute, temperature of 36.8°C, and oxygen saturation of 99% on room air. Physical examination was notable only for mild distress. Laboratory evaluation revealed a high-sensitivity troponin of 13 ng/L, normal creatine kinase-MB, mild leukocytosis (11.74 K/µL), baseline anemia (hemoglobin 11.4 g/dL), hypokalemia (3.2 mmol/L), and low bicarbonate (19 mmol/L), attributed to gastrointestinal losses.

**Figure 4:**
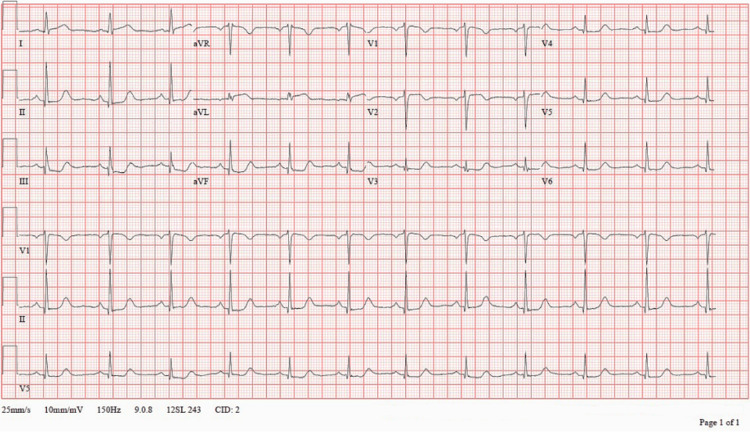
Twelve-lead electrocardiogram obtained on ED presentation demonstrating normal sinus rhythm at 70 beats per minute with mild ST-segment elevation in the lateral leads (I and aVL) and reciprocal ST-segment depression in the inferior leads (III and aVF). These dynamic changes were new compared with the patient’s ECG obtained one week earlier (Figure 2) and, in the setting of recurrent chest pain, represented a critical decision point prompting repeat echocardiography and urgent cardiology consultation for coronary angiography. Subsequent angiography confirmed spontaneous coronary artery dissection.

A repeat 12-lead ECG demonstrated normal sinus rhythm at 70 beats per minute with mild ST-segment elevation in leads I and aVL and reciprocal ST-segment depression in leads III and aVF, concerning for lateral wall ischemia (Figure 4). These changes were new compared with the tracings obtained one week earlier.

Repeat transthoracic echocardiography demonstrated newly reduced left ventricular systolic function, with an estimated ejection fraction of 45%-50% and hypokinesis of the anterior and anterolateral walls (Video 2). Given these dynamic findings, cardiology was consulted for urgent coronary angiography.

**Video 2:** Transthoracic echocardiogram in the apical four-chamber view demonstrating mildly reduced left ventricular systolic function (ejection fraction ~ 45%-50%), with hypokinesis of the anterior and anterolateral walls. The remaining walls demonstrate preserved motion, and there is no pericardial effusion or valvular abnormality. Findings are consistent with new regional wall motion abnormality due to myocardial ischemia from spontaneous coronary artery dissection (SCAD).

**Figure 5:**
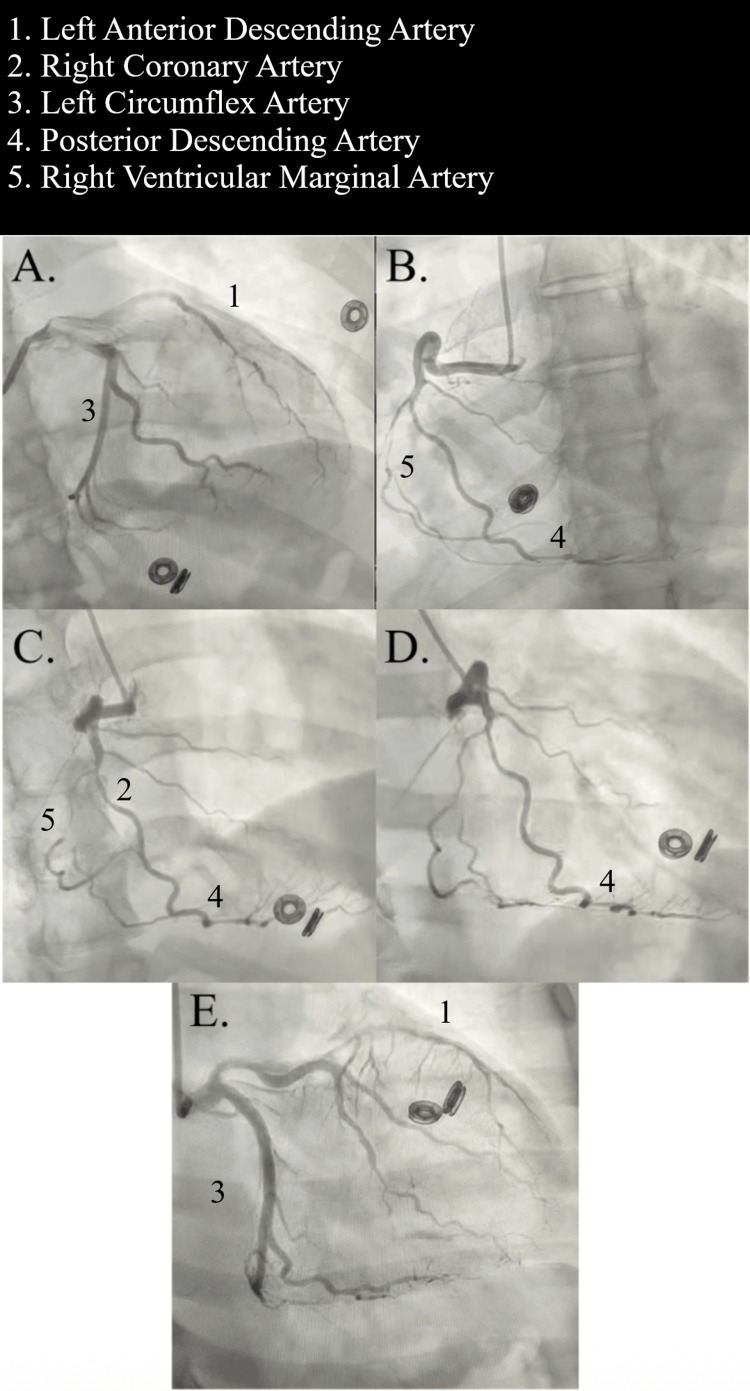
Coronary angiography demonstrating spontaneous coronary artery dissection (SCAD) of the mid-to-distal right coronary artery. (A) Right anterior oblique view of the left coronary system highlighting the left main coronary artery with luminal irregularity and smooth tapering. (B) Left anterior oblique projection of the right coronary artery confirming long, tubular narrowing consistent with type 2 SCAD. (C, D) Additional projections of the right coronary artery demonstrating the dissected segment extending distally without evidence of atherosclerotic plaque or thrombus. (E) Final frame of the left coronary system highlighting the left main coronary artery with preserved distal perfusion and TIMI 3 flow. SCAD: Spontaneous coronary artery dissection; TIMI: Thrombolysis in myocardial infarction.

Diagnostic coronary angiography revealed type 2 spontaneous coronary artery dissection involving both the right coronary artery and the left anterior descending artery, confirming non-atherosclerotic multivessel SCAD as the etiology of ACS. The right coronary artery demonstrated long, smooth, tapered narrowing extending from the mid to distal segments with preserved distal thrombolysis in myocardial infarction (TIMI) 3 flow and no evidence of atherosclerotic plaque (Figure 5, Panels A-E). The left anterior descending artery showed diffuse dissection with a diminutive true lumen and a critical proximal-to-mid stenosis, while the left circumflex artery remained patent distally. Left ventricular end-diastolic pressure was elevated at 18 mmHg. The absence of focal plaque rupture, the presence of long smooth luminal narrowing, and preserved distal TIMI 3 flow were key angiographic features confirming type 2 SCAD and directly guided the decision to pursue conservative medical management rather than percutaneous intervention.

Accordingly, no percutaneous coronary intervention was performed. An intra-aortic balloon pump was placed for management of ongoing angina, and the patient was initiated on dual antiplatelet therapy (aspirin and ticagrelor), anticoagulation, beta-blocker therapy, and high-intensity statin therapy. She was transferred to a tertiary care center for multidisciplinary cardiac management and proactive cardiothoracic surgical evaluation in the event of clinical deterioration. The patient stabilized on medical therapy and was subsequently discharged home.

At the two-week outpatient follow-up, the patient remained clinically stable. Electrocardiography demonstrated a normal sinus rhythm with a septal infarct pattern and a heart rate of 77 beats per minute, without evidence of acute ischemia. She continued dual antiplatelet therapy with aspirin 81 mg and clopidogrel 75 mg daily, high-intensity statin therapy with atorvastatin 40 mg daily, and metoprolol succinate extended-release at a dose of 25 mg three times daily. She denied recurrent chest pain or other cardiac symptoms. Counseling emphasized lifestyle modification and adherence to medical therapy, with plans for continued close cardiology follow-up and outpatient evaluation for associated arteriopathies. A summarized timeline of key clinical events, diagnostic findings, and management decisions is provided in Table 1.

**Table 1:** Timeline of clinical events, diagnostic findings, and management decisions This table outlines the patient’s sequential clinical course from the initial ED presentation through coronary angiography. Early findings during the observation-unit evaluation were nonischemic and supported discharge. Subsequent recurrence of symptoms with dynamic electrocardiographic changes and new left ventricular systolic dysfunction prompted cardiology consultation and urgent angiography, which confirmed multivessel spontaneous coronary artery dissection and guided conservative management. TTE: Transthoracic echocardiography; PVCs: Premature ventricular contractions; EMS: Emergency medical services; LV: Left ventricle; EF: Ejection fraction; SCAD: Spontaneous coronary artery dissection; RCA: Right coronary artery; LAD: Left anterior descending artery; ED: Emergency department.

Timepoint	Key Findings	Diagnostic Data	Clinical Decision
Initial ED visit (Day 7)	Edema, hypertension, no chest pain	Mild HS-troponin elevation with downtrend; normal ECG; normal TTE	Observation-unit discharge
EMS presentation (Day 0)	Acute chest pain, dyspnea, vomiting	PVCs on ECG	ED transport
ED arrival	Recurrent ischemic symptoms	Lateral ST elevation with reciprocal inferior changes	Cardiology consultation
Repeat TTE	New LV dysfunction	EF 45%-50%, anterior hypokinesis	Urgent angiography
Angiography	Multivessel SCAD	Type 2 RCA and LAD dissection	Conservative management

## Discussion

Spontaneous coronary artery dissection is increasingly recognized as a leading cause of myocardial infarction in women under the age of 50 and frequently occurs in the absence of traditional atherosclerotic risk factors [5-7]. Despite growing awareness, SCAD remains diagnostically challenging because its clinical presentation overlaps with that of classic ACS, while standard ED risk stratification tools are not designed to detect non-atherosclerotic pathology [1,5,8]. Early symptoms may resolve, biomarkers may be nondiagnostic or downtrending, and initial electrocardiographic or echocardiographic findings may appear reassuring.

Observation-unit chest pain protocols, such as those derived from the HEART pathway or Emergency Department Assessment of Chest Pain Score (EDACS), are highly effective in identifying patients at low risk for atherothrombotic ACS using structured symptom assessment, ECG interpretation, and serial high-sensitivity troponin testing [1-4]. However, these pathways may not reliably identify evolving SCAD, particularly when early symptoms resolve, biomarkers downtrend, and initial imaging is reassuring. Although the patient appropriately met institutional low-risk criteria during her initial presentation, she developed clinically significant multivessel SCAD days later. The subsequent emergence of lateral ST-segment elevation with reciprocal inferior changes (Figure 4) represented a key diagnostic inflection point, signaling active myocardial ischemia and prompting escalation beyond algorithm-based reassurance.

This case highlights a clinically important inflection point for emergency physicians: re-presentation with ischemic symptoms shortly after recent observation-unit discharge, especially when accompanied by dynamic ECG changes or evolving ventricular dysfunction. In such scenarios, reliance on prior negative testing may be inappropriate, and renewed diagnostic evaluation, including repeat ECGs, echocardiography, and early cardiology consultation, should be strongly considered, even in younger patients without classic cardiovascular risk factors. While accelerated diagnostic pathways improve efficiency and safety at a population level, this case illustrates the necessity of clinical judgment when patient trajectories deviate from expected algorithmic outcomes.

Management of SCAD differs fundamentally from that of atherothrombotic ACS. In hemodynamically stable patients, conservative medical management is preferred due to high rates of spontaneous vessel healing and the elevated risk of procedural complications associated with percutaneous coronary intervention in fragile, dissected vessels [5,9,10]. In this case, angiographic confirmation of diffuse type 2 SCAD with preserved distal TIMI 3 flow (Figure 5, Panel E) directly informed the decision to defer PCI in favor of conservative medical management. Short-term dual antiplatelet therapy and statin therapy were initiated due to multivessel involvement, ongoing ischemic symptoms, and diagnostic uncertainty at presentation. While routine statin therapy is not recommended in isolated SCAD without dyslipidemia or atherosclerosis, these agents are commonly used during the initial ACS phase and reassessed once the diagnosis is clarified [5,6,9].

Evaluation for associated extracoronary arteriopathies, including fibromuscular dysplasia, is recommended following a diagnosis of SCAD and was planned as part of this patient’s outpatient follow-up and long-term risk stratification [5,11]. Given reported recurrence rates of 10%-20%, patient education, avoidance of precipitating stressors, and longitudinal cardiology follow-up remain essential components of care [11]. The absence of pregnancy-associated triggers, known connective tissue disease, or prior vascular abnormalities in this patient further underscores why suspicion for SCAD can be particularly challenging within standard ED risk stratification frameworks.

For emergency physicians, this case reinforces that accelerated chest pain pathways should be viewed as decision-support tools rather than definitive exclusions of all serious pathology. When patients re-present with concerning symptoms after recent discharge, particularly with new ECG or echocardiographic abnormalities, clinicians should prioritize reassessment over reassurance. Although limited by its single-case nature, this report highlights a critical diagnostic gap and underscores the importance of recognizing when clinical judgment must supersede algorithm-based decision-making to ensure timely diagnosis and optimal outcomes.

## Conclusions

This case underscores the importance of considering SCAD in young women presenting with recurrent ischemic symptoms, even after a negative observation-unit evaluation conducted using contemporary chest pain pathways. While accelerated diagnostic algorithms improve efficiency and safety for atherothrombotic disease, they may not reliably detect evolving non-atherosclerotic causes of ACS. Re-presentation with chest pain, dynamic ECG changes, or new ventricular dysfunction should prompt clinicians to override prior algorithmic reassurance and pursue renewed evaluation. Although limited by its single-case nature, this report highlights a critical diagnostic inflection point where clinical judgment must supersede pathway-based decision-making to ensure timely diagnosis and optimal outcomes.
